# Somatosensory Cortex Repetitive Transcranial Magnetic Stimulation and Associative Sensory Stimulation of Peripheral Nerves Could Assist Motor and Sensory Recovery After Stroke

**DOI:** 10.3389/fnhum.2022.860965

**Published:** 2022-04-11

**Authors:** Aristela de Freitas Zanona, Andressa Claudia Romeiro da Silva, Adriana Baltar do Rego Maciel, Livia Shirahige Gomes do Nascimento, Amanda Bezerra da Silva, Nadia Bolognini, Katia Monte-Silva

**Affiliations:** ^1^Applied Neuroscience Laboratory, Universidade Federal de Pernambuco, Recife, Brazil; ^2^Department of Psychology, University of Milano Bicocca, Milan, Italy; ^3^Neuropsychological Laboratory, IRCCS Istituto Auxologico Italiano, Milan, Italy

**Keywords:** rTMS, somatosensory cortex, stroke, occupational therapy, physical therapists, neurological rehabilitation

## Abstract

**Background:**

We investigated whether transcranial magnetic stimulation (rTMS) over the primary somatosensory cortex (S1) and sensory stimulation (SS) could promote upper limb recovery in participants with subacute stroke.

**Methods:**

Participants were randomized into four groups: rTMS/Sham SS, Sham rTMS/SS, rTMS/SS, and control group (Sham rTMS/Sham SS). Participants underwent ten sessions of sham or active rTMS over S1 (10 Hz, 1,500 pulses, 120% of resting motor threshold, 20 min), followed by sham or active SS. The SS involved active sensory training (exploring features of objects and graphesthesia, proprioception exercises), mirror therapy, and Transcutaneous electrical nerve stimulation (TENS) in the region of the median nerve in the wrist (stimulation intensity as the minimum intensity at which the participants reported paresthesia; five electrical pulses of 1 ms duration each at 10 Hz were delivered every second over 45 min). Sham stimulations occurred as follows: Sham rTMS, coil was held while disconnected from the stimulator, and rTMS noise was presented with computer loudspeakers with recorded sound from a real stimulation. The Sham SS received therapy in the unaffected upper limb, did not use the mirror and received TENS stimulation for only 60 seconds. The primary outcome was the Body Structure/Function: Fugl-Meyer Assessment (FMA) and Nottingham Sensory Assessment (NSA); the secondary outcome was the Activity/Participation domains, assessed with Box and Block Test, Motor Activity Log scale, Jebsen-Taylor Test, and Functional Independence Measure.

**Results:**

Forty participants with stroke ischemic (*n* = 38) and hemorrhagic (*n* = 2), men (*n* = 19) and women (*n* = 21), in the subacute stage (10.6 ± 6 weeks) had a mean age of 62.2 ± 9.6 years, were equally divided into four groups (10 participants in each group). Significant somatosensory improvements were found in participants receiving active rTMS and active SS, compared with those in the control group (sham rTMS with sham SS). Motor function improved only in participants who received active rTMS, with greater effects when active rTMS was combined with active SS.

**Conclusion:**

The combined use of SS with rTMS over S1 represents a more effective therapy for increasing sensory and motor recovery, as well as functional independence, in participants with subacute stroke.

**Clinical Trial Registration:**

[clinicaltrials.gov], identifier [NCT03329807].

## Introduction

Upper-limb (UL) motor disability is a common problem in most stroke survivors, which substantially impacts rehabilitation costs (Feigin et al., 2014; Benjamin et al., 2018; Schwarz et al., 2019). Consequently, researchers have spent considerable time and money establishing UL therapies (Pollock et al., 2014; Hatem et al., 2016). The ability to perform effective and functional movements depends on the ability to perceive textures and object characteristics accurately and discriminate sensations of pressure, temperature, pain, vibration, and the location of body parts (Borich et al., 2015; Umeda et al., 2019). However, although more than 60% of post-stroke participants have sensory deficits, the assessment and treatment of sensation remain poorly understood in the clinical setting (Connell et al., 2008; Schabrun and Hillier, 2009; Carlsson et al., 2018), and somatosensory disorders contribute to motor disability combined with injury or neurological disorders (Brodie et al., 2014; Borich et al., 2015; Umeda et al., 2019).

Despite growing evidence showing that sensory stimulation, even without motor training, may be beneficial for post-stroke sensory and motor impairments (Cambier et al., 2003; Bolognini et al., 2016), intervention protocols or post-stroke functional recovery are still unclear (Carlsson et al., 2018).

Recently, non-invasive brain stimulation (NIBS) has been investigated as a tool that can act synergistically with conventional therapies, thereby enhancing its effects on neurological rehabilitation (Nitsche et al., 2008; Rossi et al., 2012). NIBS is painless, safe, and has the potential to modulate central nervous system excitability and induce plasticity (Boros et al., 2008; Rossi et al., 2012; Lefaucheur et al., 2014). Among these techniques, repetitive transcranial magnetic stimulation stands out. Although repetitive transcranial magnetic stimulation (rTMS) over the primary motor cortex (M1) seems to be a safe and effective alternative strategy to promote UL recovery in stroke (Dionísio et al., 2018), the efficacy of rTMS over the primary somatosensory cortex (S1) still needs to be investigated (Leodori et al., 2017). Likewise, it is still unclear whether related central therapies, such as rTMS, with peripheral therapies, can have superior effects on upper limb recovery.

Previous evidence in healthy participants showed that S1 stimulation by 5 Hz rTMS increases somatosensory cortical excitability (Ragert et al., 2004) and drives functional reorganization of cortical maps along with increased tactile acuity (Pleger et al., 2004). In contrast, 1 Hz rTMS over S1 impairs somatosensation and decreases motor skill acquisition (Vidoni et al., 2010). These findings suggested that S1 may be an alternative target for rTMS in stroke rehabilitation. Indeed, a previous study in chronic stroke survivors showed that 5 Hz rTMS over S1 paired with motor practice enhanced motor learning (Brodie et al., 2014). Similarly, different types of sensory stimulation (SS), such as peripheral nerve stimulation, mirror therapy, and active sensory training, increase corticospinal excitability (Wu et al., 2005), enlarge M1 body representations (Ridding et al., 2000; Garry et al., 2005), and promote motor recovery (Brodie et al., 2014). For a more detailed review of sensory stimulation, refer to Schabrun and Hillier (2009) and Serrada et al. (2019).

In addition, given the evidence that the adjuvant use of rTMS may enhance motor training effects (Du et al., 2016), combining rTMS over S1 with SS might promote larger clinical gains than the individual single therapies of SS or rTMS. The use of different sensory strategies has been encouraged in recent literature to treat sensorimotor deficits, as multisensory stimulation through exposure to an enriched environment increases brain plasticity and recovery of function after stroke (Hakon et al., 2018). This type of multisensory stimulation may affect sensorimotor bodily representations at varying levels, thereby optimizing perception and motor behavior (Bolognini et al., 2015; Tinga et al., 2016; Sathian and Ramachandran, 2020).

Therefore, through clinical measures based on the domains of the International Classification of Functioning, Disability, and Health (ICF), this preliminary study aimed to investigate whether rTMS over S1 and SS might promote UL recovery (ICF Body-Structure/Function and Activity/Participation domains) in subacute stroke survivors and whether the combination of rTMS with SS can induce larger improvements as compared to their individual administration.

Compared with previous studies conducted in the chronic stage of stroke, this study focused on the subacute stroke phase. Early (7 days to 3 months) and late (3 months to 6 months) subacute periods (Bernhardt et al., 2017) represent a more appropriate time for rehabilitation, and treatments in this stage of illness may increase and guide optimal spontaneous reorganization of motor networks, thereby facilitating the functional recovery process (Stinear and Byblow, 2014; Zhang et al., 2017). This period is also characterized by a greater demand for outpatient rehabilitation services by both the patient and family.

Therefore, we hypothesize that combining rTMS in S1 therapy with active sensory therapies may not only be a promising strategy to improve upper limb recovery but also activity and participation. Thus, we aimed to investigate whether rTMS over the primary somatosensory cortex (S1) and sensory stimulation (SS) could promote upper limb sensorimotor function and to increase such a person’s activity and participation in activities of daily living in participants with subacute stroke.

Our main analyses focused on ICF body structure/function outcomes and secondary analyses on activity/participation outcomes.

## Materials and Methods

### Trial Design

This sham-controlled, triple-blind, randomized pilot study was conducted at the Applied Neuroscience Laboratory at the Universidade Federal de Pernambuco. The trial was approved by the local Human Research Ethics Committee and registered in the Clinical Trials database (NCT03329807).^1^

### Participants

Stroke survivors were recruited from outpatient clinics and university hospitals through advertisements between November 2017 and April 2019. The inclusion criteria were adults (aged 30–75 years) with hemiparesis (UL Fugl-Meyer assessment, UL-FMA score between 10 and 62) due to ischemic or hemorrhagic stroke confirmed by imaging examinations. Participants underwent imaging examinations at the time of screening, in addition to reports and previous examinations. The participants were in the subacute stage of the illness (early and late, from 3 to 24 weeks). The exclusion criteria were as follows: Mini-Mental State Examination (MMSE) (Folstein et al., 1975) score <18; history of multiple brain lesions, other associated neurological diseases, peripheral sensory disorders, psychiatric disorders (including drug and alcohol abuse), UL deformities, contraindications to rTMS (Rossi et al., 2009), and intake of drugs affecting cortical excitability. Participants who were unable to perceive transcutaneous electrical neurostimulation (TENS) on the hand and forearm or were undergoing another concurrent UL treatment were also excluded.

### Randomization and Blinding

Participants were randomized into four groups after baseline assessment: (i) rTMS/sham SS, (ii) sham rTMS/SS, (iii) rTMS/SS, and (iv) control (sham rTMS and sham SS). A stratified block allocation based on stroke onset and patient age was performed by an independent researcher at Randomization.com.^2^ The group assignment was enclosed in sequentially numbered, opaque, sealed envelopes. The double-blind study masked the allocation of the intervention to participants and evaluators who were blinded to the treatment arm. Each table represents different strata, in which participants were allocated according to their age and stroke time, namely: stratum 1 with 3 and 12 weeks elapsed from the stroke, aged between 30 and 55 years; stratum 2, from 13 to 24 weeks after the stroke, aged 56–75 years; stratum 3, between 13 and 24 weeks after the stroke, aged 30–55 years; and stratum 4, between 3 and 12 weeks after the stroke, aged 56–75 years. We decided to stratify participants beforehand because age and stroke stage could interfere with the treatment effects.

These extracts served to ensure that all four groups were homogeneous in terms of age and stroke onset (weeks). In all groups, we had the same number of people with similar post-stroke duration and similar ages that could be compared with each other.

**FIGURE 1:**
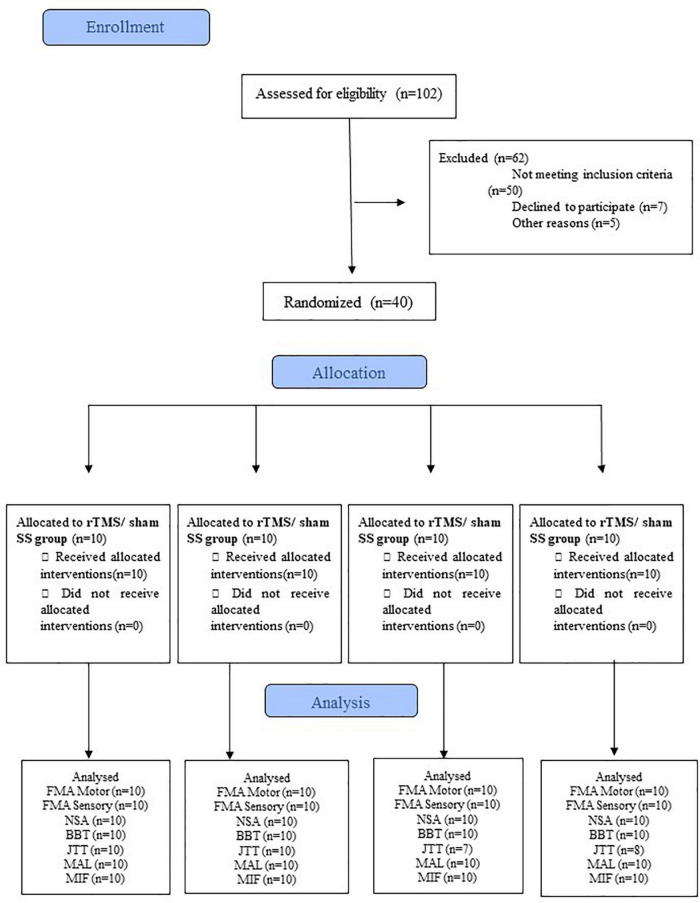
CONSORT flowchart of the study. BBT, Box and Block test; FIM, Functional Independence Measure; FMA-Motor, upper limb Fugl-Meyer for motor function; FMA-Sensory, upper limb Fugl-Meyer assessment for sensory function; JTT, Jebsen-Taylor Hand Function Test; MAL, Motor Activity Long test;. NSA, Nottingham Sensory Assessment; SS, Sensory Stimulation. rTMS, repetitive Transcranial Magnetic Stimulation.

Each treatment lasted for 10 days (2 weeks of treatment, 5 days per week, from Monday to Friday). A flowchart describing the participants at each stage of the trial is shown in Figure 1.

### Outcome Measures

Clinical assessment was performed by trained staff before and at the end of the 10-day treatment period. Assessments were performed 1 day before the start of treatment, and a reassessment was performed the day after the end of the 10-day protocol/sessions. Our main analyses focused on the ICF body structure/function outcomes and secondary analyses of activity/participation outcomes.

### Primary Outcomes–International Classification of Functioning, Disability, and Health Body Structure/Function Domains

#### Motor and Sensory Functions

The UL-FMA was used to assess motor (FMA-motor) and sensory (FMA-sensory) functions. The FMA-motor score ranged from 0 to 66, and the FMA-sensory score ranged from 0 to 12 (Fugl-Meyer et al., 1975; Maki et al., 2006). The minimal clinically important difference (MCID) for UL-FMA was 6.6 and 1.2 points (10% of total scores) for motor and sensory functions, respectively (Gladstone et al., 2002).

The Nottingham Sensory Assessment (NSA) evaluates tactile sensation (light touch, pressure, pinprick, temperature, tactile location on both sides of the body, and simultaneous bilateral touch), stereognosis, proprioception, and two-point discrimination. During the evaluation, the participants were blindfolded, and the room temperature was maintained constant. Each NSA item was graded as 0 (absent), 1 (altered sensation), or 2 (normal sensation), except for proprioception, which was scored from 0 (absence) to 3 (normal proprioception). The total NSA score ranged from 0 to 108 for the affected side (Lima et al., 2010).

### Secondary Outcomes—International Classification of Functioning, Disability, and Health Activity/Participation Domains

#### Manual Dexterity

The Box and Block Test (BBT) was used to evaluate manual dexterity. During the BBT, participants were instructed to transfer wood blocks (2.5 cm) with their paretic hand as fast as possible from one side of a compartment to the other within 60 s (Kontson et al., 2017). For stroke participants, the BBT MCID was 5.5 blocks per minute (Chen et al., 2009).

#### Arm Function

The Motor Activity Log (MAL) scale was used to measure quality (MAL-QOM) and amount of UL movement (MAL-AOM) in daily activities. For each activity, participants reported how much (quantity of movement) and how well (quality of movement) the activity was performed on a six-point scale, ranging from 0 (worst performance) to 5 (best performance) (Pereira et al., 2012). In MAL-QOM and MAL-AOM, the MCID was 1.0 points for the paretic non-dominant limb or 1.1 points for the paretic dominant limb (Lang et al., 2008).

The Jebsen-Taylor test (JTT) was used to measure the time required to complete six tasks with the paretic hand: turning over cards, picking up small common objects, simulated feeding, stacking checkers, and moving five light and heavy cans (Ferreiro et al., 2010). Participants who could not perform the test within 120 seconds were excluded from the analysis.

#### Functional Independence

Functional independence in six domains (personal care, sphincter control, mobility, locomotion, communication, and social cognition) was assessed using the Functional Independence Measure (FIM). Each domain is scored from 1 (total assistance) to 7 (complete independence). The total FIM score ranges from 18 (lowest; i.e., total dependence) to 126 (highest; i.e., complete independence) (Riberto et al., 2004). The MCID for the FIM was 22 points (Beninato et al., 2006).

### Interventions

#### Repetitive Transcranial Magnetic Stimulation

**FIGURE 2:**
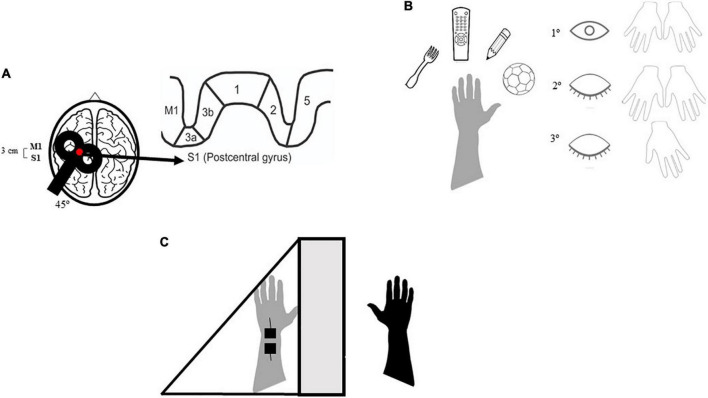
Demonstration of the therapeutic environment of repetitive transcranial magnetic stimulation (rTMS) and sensory stimulation. M1, primary motor cortex; S1, primary somatosensory córtex. Participants who were part of the active rTMS/SS group received 20 min of rTMS over S1 followed by the active sensory training: the participant has to haptically explore different textures of familiar objects and to recognize their different characteristics. First with both hands (intramodal calibration) and eyes open (cross-modal calibration) then using only the affected hand and eyes closed (Carey et al., 2011). Firstly, we required to explore the object with eyes closed and both hands, then with only the affected hand and eyes closed. The final part of the training consisted in Mirror Therapy in addition to transcutaneous electrical nerve stimulation (TENS). **(A)** rTMS on S1, 20 min. **(B)** Active sensory training, 20–25 min. **(C)** Mirror therapy/TENS, 40–45 min.

A Magstim rapid stimulator (The MAGSTIM^®^ Company LTD–United Kingdom) connected to a 70 mm figure-of-eight coil was used for the rTMS application. Participants received ten daily applications of sham or active rTMS (30 trains at 10 Hz, each lasting 5 s with an inter-train interval of 30 s) over the S1 of the lesioned hemisphere. S1 was delimited at a point 3 cm posterior to the hotspot of the first dorsal interosseous muscle (FDI) of the paretic hand. The hotspot was defined as the location where the largest and most consistent visual responses were elicited by single-pulse TMS for the FDI muscle. If an FDI hotspot was not found, S1 was delimited at a point 3 cm posterior to M1 and localized at C3/C4 of the 10–20 EEG system (Figure 2). Previous studies had shown changes in sensory function when non-invasive brain stimulation was applied 3 cm posterior to the hand area of the primary motor cortex (Fiorio and Haggard, 2005; Koch et al., 2006). The stimulation intensity was set at 120% of the individual resting motor threshold (RMT) for the FDI of the non-lesioned hemisphere (Wupuer et al., 2013; Milot et al., 2019). RMT was defined as the lowest magnetic pulse stimulus intensity required to elicit a visual twitch in five of ten trials in at least one of the contralateral hand resting muscles. This method is a safe, accurate, and reliable technique for obtaining RMTs (Varnava et al., 2011), and it was adopted because the repetitive-pulse magnetic stimulator (Magstim super rapid) was not connected to an EMG system. RMT was assessed on each day of stimulation. As previously reported by other authors (Wupuer et al., 2013; Milot et al., 2019), the non-lesioned hemisphere RMT was used to determine the rTMS intensity. A substantial portion of the M1 or corticospinal tract is usually damaged after a stroke and causes the RMT of the lesioned hemisphere to increase substantially, indicating that the stimulator output would not have reached 120% of the RMT for all subjects (Wupuer et al., 2013; Milot et al., 2019).

For sham-rTMS, a 70 mm figure-of-eight coil was held over S1 while disconnected from the stimulator, and rTMS noise was presented during the sham stimulation session with two computer loudspeakers positioned behind the participant (out of their view). rTMS noise was created by recording an active rTMS session. Thus, while no magnetic pulses were delivered to the participant with sham rTMS, they were exposed to the same noise as the active protocol.

None of the participants had undergone neuromodulation previously and were thus unaware of the operation of the coil and repetitive TMS. Although the patient felt the stimulus made by the coil to establish the resting motor threshold, this stimulus was very different from the stimulus of repetitive TMS therapy. Thus, the patient was informed that the assessment differed from the intervention. At the end of each session, an adverse effects questionnaire was administered to each patient. In this questionnaire, the participant also answered whether he believed that he had received real or fictitious stimulation. In response, 92.5% of participants believed that they had received real stimulation.

#### Sensory Stimulation

Immediately after the rTMS sessions, participants received active or sham SS. To target the somatosensory system at different levels, the SS protocol comprised 20–25 min of active sensory training, 40 min of mirror therapy (i.e., visual-proprioceptive stimulation), and 45 min of transcutaneous electrical nerve stimulation (TENS). Combining information from different sensory modalities (visual, proprioceptive, and somatosensory) can be especially beneficial in supporting perception and action when unimodal information is weakly effective, as it may occur when a stroke affects somatosensory processing (Sathian and Ramachandran, 2020).

Active sensory training consisted of sensory discrimination training, as described by Carey et al. (2011), divided into four sensory tasks: texture discrimination, graphesthesia, limb position sense, and tactile object recognition. The texture-discrimination task employed graded stimuli with varying sensory features (texture, shape, size, weight, and hardness). In the graphesthesia task, participants were asked to identify a series of numbers, letters, and geometric shapes drawn on the palmar and dorsal surfaces of the hand using a pencil. For the limb position sense task, the upper limb of the participant was moved to a position in the flexion-extension, abduction-adduction, and pronation-supination ranges; participants had to report the perceived position of their limb. In the tactile object recognition task, the researcher asked the blindfolded participant to reach and remove objects from a basket placed in front of them in a random sequence. The tasks were performed first using the non-paretic hand and then with the paretic hand (with or without visual feedback). Active sensory training was performed only with the non-paretic hand in the sham SS group.

For mirror therapy, the paretic upper limb was hidden behind a mirror (50 × 50 cm), placed at the center of a table in front of the subject, and the non-paretic upper limb was placed in front of the mirror. Participants were asked to look at the non-paretic upper limb reflected in the mirror and observe the movements (flexion-extension of the wrist, elbow, and fingers, and pronation-supination of the forearm) (Cho and Cha, 2015). In the sham SS group, non-paretic UL limb movements were executed without mirror reflection.

Transcutaneous electrical nerve stimulation (TENS) (Dualpex 961—Quark^®^ medical products) was delivered using a surface electrode placed on the median nerve at the wrist of the paretic upper limb. The application of TENS to the median nerve, performed during MRI in healthy subjects, has been shown to activate the main sensory regions of the brain in the hemisphere contralateral to stimulation (Kampe et al., 2000).

TENS was used in this study to the detriment of Neuromuscular Electrical Stimulation (NMES), as TENS is an alternative way of electrical stimulation that has historically been used at high frequencies for pain relief (DeSantana et al., 2008) and spasticity (Armutlu et al., 2003), but it is now also administered at very low frequencies (sensory level 2–10 Hz) for the purpose of improving sensory function. The NMES, on the other hand, is typically provided at higher frequencies (20–50 Hz) expressly to produce tetany and muscle contraction (Doucet et al., 2012).

Sensory TENS has been growing interest in the past decade in its use as a means of improving recovery sensory and motor after a stroke (Laufer and Elboim-Gabyzon, 2011).

The stimulation intensity was determined as the minimum intensity at which the participants reported paresthesia (no muscle contraction). First, the intensity was increased until the researcher perceived the muscle contraction by palpation (motor threshold) and then the intensity was reduced to only provoke paresthesia in the arm and hand (sensory threshold). Five electrical pulses (1 ms duration each) at 10 Hz were delivered every second for 45 min (Conforto et al., 2010). The participants were instructed not to make muscle contractions during the stimulation. Peripheral nerve sensory stimulation was performed concomitantly with mirror therapy. During the sham stimulation, the device was turned off 60 s after the stimulation onset to mimic the initial skin sensations.

During the intervention, the sham SS participants were convinced that training the unaffected hand could serve as a parameter for the affected hand to relearn the movements.

In general, participants in the active SS group received a sensory therapy protocol that consisted of 20–25 min of active sensory training and 40 min of mirror therapy, concomitant with 45 min of TENS (Figure 2).

Participants in the sham SS group received active sensory training in the non-paretic hand. The entire set of mirror therapy tasks was performed as non-paretic upper limb movements without a mirror. For TENS for the sham stimulation, the device was turned off for 60 s after stimulation onset, giving only the initial sensation of stimulation without actually occurring. Participants were convinced that they could no longer feel the electrical stimulus due to habituation of the sensory system.

The control group did not participate in any other therapies for the duration of the study. Participants and family members were asked to take a break from other treatments, and if the patient remained on another therapy, they were discontinued from the study.

### Data Processing and Analysis

We evaluated whether the data were normally distributed using the Shapiro–Wilk test. One-way analysis of variance (ANOVA; normal data distribution) or Kruskal–Wallis (non-normal distribution) for continuous variables, and Fisher’s exact test for categorical variables were used to analyze differences in baseline characteristics among the four groups.

Given the non-normal distribution of post-intervention data of primary outcomes, non-parametric statistics (Kruskal–Wallis *U*-test) were used. Within-group and between-group comparisons were assessed using Wilcoxon signed and Mann–Whitney tests, respectively. Pre-post differences were considered for the analysis of secondary outcome measures because of differences among the groups at baseline. Furthermore, to determine the magnitude of effect for practical concern, the difference between the means of pre-and post-test divided by the standard deviation (SD) at pre-test for each group was calculated. The size of the feat was analyzed in a descriptive way to inform about the impact of the intervention factor, be it rTMS and SS, or by the association of the mean and standard deviation of the results of each outcome in each group. The results were interpreted according to Cohen (Nakagawa and Cuthill, 2007) as trivial for *d* < 0.20, small for 0.20 ≤ *d* < 0.50, moderate for 0.50 ≤ *d* < 0.80, and large for *d* ≥ 0.80.

The chi-square test was used to compare between-group differences in the proportion of participants who reached the MCID values. The MCID was analyzed descriptively to understand which therapy was superior to help the participant achieve the minimum difference expected for each assessment instrument.

Statistical Package for Social Sciences (SPSS) version 18^®^ software was used for all statistical analyzes, and a significance level of *p* ≤ 0.05 was adopted.

## Results

The entire sample (*N* = 40, ten participants in each group) had a mean age of 62.2 ± 9.6 years; as shown in Table 1, there were no differences between groups with respect to sex (*p* = 0.94), type of stroke (*p* = 0.55), the time elapsed from stroke (*p* = 0.66), the severity of hemiparesis (*p* = 0.59), manual dexterity (*p* = 0.38), cognitive level (MMSE score, *p* = 0.64), motor and sensory FMA scores (*p* = 0.19 and *p* = 0.15, respectively), NSA score (*p* = 0.67), and FIM score (*p* = 0.33). Instead, the four groups differed at baseline with respect to the ICF activity/participation domains (Table 1). For this reason, such differences between the groups were corrected by analyzing the difference before and after intervention (i.e., Δ post-treatment/baseline). No adverse events were reported by any of the participants. Five participants (three from the sham rTMS/SS group and two from the control group) could not perform the JTT and were excluded from the analysis (see Figure 1).

**TABLE 1:** Demographic and stroke characteristics for each group at baseline.

	rTMS/sham SS (*n* = 10)	Sham rTMS/SS (*n* = 10)	rTMS/SS (*n* = 10)	Control (*n* = 10)	*p*-value
Age, mean (SD)	62.6 ± 7.9	62.1 ± 11.4	62.6 ± 7.8	61.6 ± 11.3	0.988^b^
Gender, male *n* (%)	5 (50)	6 (60)	5 (50)	3 (30)	0.939^a^
Stroke Type, ischemic *n* (%)	9 (90)	10 (100)	9 (90)	10 (100)	0.551^a^
Stroke onset (weeks) mean (SD)	10.1 ± 6.6	8.9 ± 5.1	12.3 ± 6.3	11.4 ± 6.3	0.658^b^
Hemiparesis, right *n* (%)	5 (50)	3 (30)	6 (60)	6 (60)	0.592^a^
Dominance, right *n* (%)	10 (100)	9 (90)	10 (100)	10 (100)	0.380^a^
MMSE mean (SD)	25.3 ± 4.2	25.3 ± 4	23.9 ± 4.1	21.7 ± 4.6	0.638^b^
FMA-Motor mean (SD)	48.4 ± 14.3	36.5 ± 19.9	46.1 ± 11.5	39 ± 14	0.186^b^
FMA-Sensory mean (SD)	8.6 ± 2.3	7.3 ± 3.1	8.2 ± 1.7	7.6 ± 1.5	0.514^b^
NSA mean (SD)	71.1 ± 8.1	60 ± 25.9	71.3 ± 10.5	63.7 ± 20.7	0.667^b^
BBT mean (SD)	34.8 ± 9.4	18.9 ± 16.1	22.1 ± 12.1	12 ± 9.4	0.002^c^
MAL-QOM mean (SD)	1.8 ± 0.8	0.9 ± 1	0.8 ± 0.6	0.5 ± 0.3	0.003^b^
MAL-AOM mean (SD)	3.2 ± 1.1	0.9 ± 0.9	0.9 ± 0.9	0.9 ± 0.5	0.001^b^
JTT mean (SD)	82.3 ± 18.6	68 ± 13.8	122 ± 58.7	170 ± 29.8	0.006^b^
FIM mean (SD)	107.4 ± 18.2	92 ± 22.3	99 ± 18.1	91.6 ± 20.6	0.300^b^

*Data are presented as the mean and standard deviation (SD) for continuous variables or as numbers and percentages for categorical variables.*

*^a^Fisher’s exact test;*

*^b^Kruskal–Wallis test;*

*^c^One-way ANOVA. The p-value represents the difference between groups. SS, Sensory Stimulation; rTMS, repetitive transcranial magnetic stimulation; BBT, Box and Block test; FIM, Functional Independence Measure; FMA, Fugl-Meyer assessment; JTT, Jebsen-Taylor Hand Function Test; MAL-QOM, the quality of upper limb movement of Motor Activity Long test; MAL-AOM, amount of upper limb movement of motor activity long test; MMSE, mini-mental state examination; NSA, Nottingham Sensory Assessment.*

### Primary Outcomes

The results related to the body structure/function domain of the ICF are presented in Table 2. Regarding motor function (FMA-motor), all groups had showed significant improvements from baseline (Wilcoxon test, *p* ≤ 0.05): rTMS/Sham SS (*p* = 0.017), Sham rTMS/SS (*p* = 0.015), and rTMS/SS (*p* = 0.005), with the exception of the control group (*p* = 0.671). The FMA-motor scores were significantly higher in the rTMS/sham SS (*p* = 0.019) and rTMS/SS (*p* = 0.002) groups, compared to the control group (Mann-Whitney test, *p* ≤ 0.05). Moreover, the rTMS/SS group had the larger effect size (rTMS/sham SS: *d* = 0.48; sham rTMS/SS: *d* = 0.50; rTMS/SS: *d* = 1.12; control group: *d* = 0.03).

**TABLE 2:** Primary Outcomes–Body structure/function domains of the International Classification of Functioning, Disability, and Health (ICF).

	rTMS/sham SS			Sham rTMS/SS			rTMS/SS			Control		
	Baseline	Post	*d*	Baseline	Post	*d*	Baseline	Post	*d*	Baseline	Post	*d*
**Motor function FMA-motor**												
1	31	41		47	66		22	40		52	55	
2	38	53		28	46		60	66		28	24	
3	56	66		54	62		49	66		29	35	
4	58	62		14	37		48	66		54	56	
5	51	66		59	63		40	55		61	63	
6	20	20		60	65		60	62		15	19	
7	61	66		19	20		49	64		36	36	
8	62	60		10	10		39	47		37	37	
9	60	60		53	50		55	64		32	32	
10	47	59		21	46		39	62		46	38	
Total (mean ± SD)	48.4 ± 14.3	55.3 ± 14.5*^#^	0.48	36.5 ± 19.9	46.5 ± 19.3*	0.50	46.1 ± 11.5	59.2 ± 9*^#^	1.12	39.0 ± 14	39.5 ± 14.2	0.03
**Sensory function FMA-sensory**												
1	11	8		10	12		5	12		8	8	
2	4	11		5	9		8	12		6	8	
3	10	12		12	12		8	12		9	9	
4	9	11		6	12		7	12		8	10	
5	11	12		4	12		10	12		9	10	
6	8	8		10	12		10	12		8	12	
7	6	12		10	10		10	12		6	6	
8	10	11		2	2		7	10		10	10	
9	7	10		8	12		10	10		6	8	
10	10	12		6	12		7	12		6	6	
Mean ± SD	8.6 ± 2.3	10.7 ± 1.5^#^	0.91	7.3 ± 3.1	10.5 ± 3.2*^#^	1,03	8.2 ± 1.7	11.6 ± 0.8*^#^	2,0	7.6 ± 1.5	8.7 ± 1.8	0.73
**NSA**												
1	65	68		76	77		54	66		80	75	
2	80	79		65	69		80	82		78	72	
3	80	82		78	81		80	82		69	72	
4	79	82		38	78		67	77		82	82	
5	69	72		77	80		76	73		78	78	
6	69	66		79	80		74	81		51	63	
7	57	82		41	45		71	80		14	14	
8	64	71		0	2		78	78		52	75	
9	68	72		71	80		81	82		62	58	
10	80	80		75	80		52	76		71	79	
Mean ± SD	71.1 ± 8.1	75.4 ± 6.2*	0.53	60 ± 25.9	67.2 ± 25.3*	0.28	71.3 ± 10.5	77.7 ± 5.1*	0.6	63.7 ± 20.7	66.8 ± 19.9	0.16

Data are presented as the mean and standard deviation. *p-value < 0.05, compared to baseline; #P-value < 0.05 compared to control group. d, Cohen’s d effect size; FMA-Sensory, upper limb Fugl-Meyer assessment for sensory function; FMA-Motor, upper limb Fugl-Meyer assessment for motor function; NSA, Nottingham Sensory Assessment; SS, sensory stimulation; rTMS, repetitive transcranial magnetic stimulation.

For the primary sensory outcomes, compared with baseline values, NAS scores increased in all groups: rTMS/Sham SS (*p* = 0.042), Sham rTMS/SS (*p* = 0.005), and rTMS/SS (*p* = 0.028), except in the control group (*p* = 0.398). The FMA-sensory scores improved only in the groups that received active SS: Sham rTMS/SS (*p* = 0.017) and rTMS/SS (*p* = 0.007) (Wilcoxon test, *p* ≤ 0.05). Compared to the control groups (Mann–Whitney test, *p* ≤ 0.05), all groups showed significant improvements in the FMA-sensory after the treatment: rTMS/Sham SS (*p* = 0.023); Sham rTMS/SS (*p* = 0.023); rTMS/SS (*p* = 0.001), but not in the NSA (Table 2). Again, the rTMS/SS group showed a larger effect size than the other groups did.

### Secondary Outcomes

Table 3 shows the results for the secondary outcomes, with the significant differences among groups for the MAL-QOM (Kruskal–Wallis test = 16.3, *p* = 0.001), MAL-AOM (Kruskal–Wallis test = 16.1, *p* = 0.001), JTT (Kruskal–Wallis test = 9.64, *p* = 0.022), and MIF (Kruskal–Wallis test = 7.93, *p* = 0.047). In the self-care domain of the FIM, there was no difference between the groups (Kruskal–Wallis *p* = 0.87). Arm function and functional independence improved only in the rTMS/SS group (Mann–Whitney test, MAL-QOM: *Z* = −3.63, *p* < 0.001; MAL-AOM: *Z* = −3.81, *p* < 0.001; JTT: *Z* = −3.63, *p* < 0.001; MIF: *Z* = −2.27, *p* = 0.023) compared to those of the control group. The quality of movement also improved in the rTMS/sham SS group, compared with that in the control group (Mann–Whitney test, *Z* = −2.62, *p* = 0.007).

**TABLE 3:** Secondary outcomes–activity/participation domains of the International Classification of Functioning, Disability, and Health (ICF).

	rTMS/sham SS		Sham rTMS/SS		rTMS/SS		Control	
	Pre-post dif.	*d*	Pre-post dif	*d*	Pre-post dif	*d*	Pre-post dif	*d*
**Manual dexterity**								
BBT	5.4 ± 6.6	0.66	5.4 ± 7.1	0.33	6.8 ± 11.0	0.56	2.7 ± 4.0	0.28
**Arm function**								
MAL-QOM	1.1 ± 1.1^#^	1.36	0.4 ± 0.9	0.44	2 ± 1.5^#^	2.67	0.05 ± 0.1	0.15
MAL-AOM	0.5 ± 0.9	0.46	0.4 ± 0.9	0.43	1.8 ± 1.4^#^	2.22	0.04 ± 0.2	0.08
JTT	−22.4 ± 21.0	1.20	−21.6 ± 18.1	1.56	−59.7 ± 44^#^	1.07	−4.0 ± 11.3	0.11
**Functional independence**								
FIM Self care	2.3 ± 2.4	0.06	7.7 ± 3.8	1.38	8.3 ± 7.8	0.68	4.2 ± 5	0.36
FIM total	3.8 ± 4.0	0.20	13.1 ± 13.2	0.59	16.6 ± 13.6^#^	0.91	3.8 ± 4.9	0.18

*Data are presented as pre-post difference (pre-post dif) mean and standard deviation. d, Cohen’s d effect size; BBT, Box and Block Test; JTT, Jebsen-Taylor Test; MAL-QOM, Motor Activity Long test-the quality of upper limb movement; MAL-AOM, Motor Activity Long test, the amount of upper limb movement; FIM, Functional Independence Measure; SS, sensory stimulation; rTMS, repetitive transcranial magnetic stimulation. #p-value < 0.05, compared with the control group.*

### Minimal Clinically Important Difference

**FIGURE 3:**
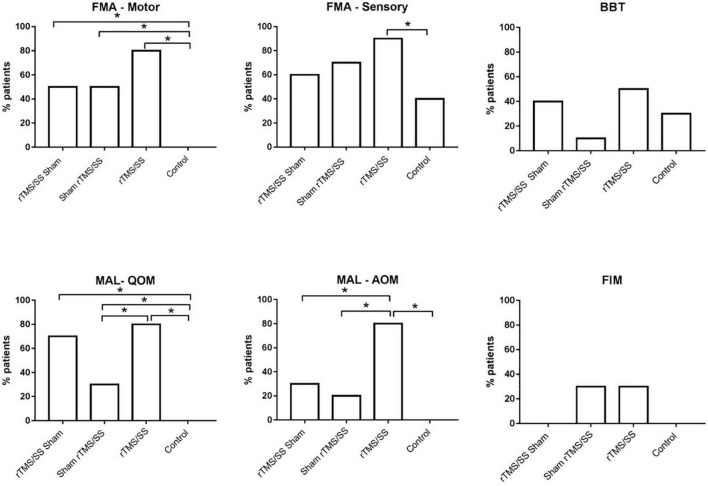
Proportion of subjects who reached the minimal clinically important difference (MCID) for each group. *p-value < 0.05 Chi square. BBT, Box and Block Test; FMA-Sensory, upper limb Fugl-Meyer assessment for sensory function; FMA-Motor, upper limb Fugl-Meyer assessment for motor function; FIM, Functional Independence Measure; MAL, Motor Activity Log; QOM, the quality of upper limb movement of Motor Activity Long test; MAL-AOM, amount of upper limb movement of Motor Activity Long test. SS, sensory stimulation; rTMS, repetitive transcranial magnetic stimulation.

The proportion of participants who reached the MCID was significantly higher in all the experimental groups than in the control group with respect to the FMA-motor and MAL-QOM; a higher proportion of participants in the rTMS/SS group reached the MCID at the FMA-sensory and MAL-AOM. A higher proportion of participants reached the MCID in the rTMS/SS group for most outcomes (Figure 3).

## Discussion

The study findings suggest that, compared to the control group, all experimental groups who received active rTMS or SS showed significant improvements in all primary outcomes (ICF Body Structure/Function domains) and some secondary outcomes (of the Activity/Participation domains of the ICF). However, stroke survivors receiving rTMS combined with SS showed greater benefits than the groups receiving only rTMS or only SS. All participants tolerated the treatment well, and no adverse events were observed.

### Effect of Monotherapy (Repetitive Transcranial Magnetic Stimulation or Sensory Stimulation)

Our study suggests that our SS protocol delivered as monotherapy only improved somatosensation (FMA-sensory). Overall, similar sensory improvements at the level of body structure and function have been previously reported after each type of SS intervention (Enders et al., 2013; Kilgard et al., 2018). However, the benefits of SS on motor outcomes at the level of body structure/function and activity/participation in stroke survivors remain to be clarified (Enders et al., 2013). Similar to our results, a systematic review with meta-analyses presented low to moderate quality evidence from clinical trials, suggesting that peripheral electrical stimulation combined with sensorimotor tasks does not improve UL motor impairment or activity more than usual (Grant et al., 2017). In our study improvements in sensory function after sham rTMS/SS were insufficiently robust to enhance motor outcomes. We were unable to demonstrate whether our SS protocols, comprising different forms of sensory stimulation, were more effective than each SS intervention. A growing body of evidence shows the clinical superiority of multisensory stimulation, which could allow the emergence of various forms of crossmodal plasticity (Johansson, 2012; Sathian and Ramachandran, 2020). Optimal multisensory integration may overcome modality-specific disorders and improve motor behavior (Bolognini et al., 2013).

To the best of our knowledge, no studies have reported the effect of rTMS over S1 on post-stroke motor and sensory impairments and the functional independence of subacute stroke survivors in daily life activities. Brodie et al. (2014) reported that 5 Hz rTMS over the lesioned S1 paired with motor practice enhanced motor learning and tactile acuity, which were both measures of the body structure/function domain, but that it did not affect functional outcomes. In our study, S1-rTMS delivered alone not only improved motor (FMA-motor) and sensory (FMA-sensory) disorders but the quality of arm movement in daily activities, as well. The difference between the rTMS protocol of Brodie’s study (5 rTMS sessions, 5 Hz, 1,200 pulses, 90% RMT) and our protocol (10 rTMS sessions, 10 Hz, 1,500 pulses, 120% RMT) may partly explain the different results.

Two main mechanisms may explain the effects induced by S1-rTMS observed in our study. First, 10 Hz rTMS would increase S1 excitability, thereby directly improving somatosensation and indirectly improving motor functions. Real-time somatosensory feedback is essential for optimal motor performance and control (Kessner et al., 2016). Considering the dense anatomo-functional connections between S1 and M1 (White and Deamicis, 1977; Donoghue and Parham, 1983; Veinante and Deschênes, 2003), another hypothesis is that ipsilesional S1-rTMS can increase the excitability of the injured M1 and facilitate sensorimotor functional recovery (Kim et al., 2006). Further studies are necessary to gain a clearer understanding of the mechanisms involved in the motor effects induced by rTMS over the S1.

### Effect of Combination Therapies (Repetitive Transcranial Magnetic Stimulation and Sensory Stimulation)

Our results point that S1-rTMS and SS combined led to greater benefits for stroke recovery in both the ICF body structure/function and activity/participation domains than the single intervention (S1-rTMS alone or SS alone). Some clinical trials have supported the advantages of combining motor therapies with non-invasive brain stimulation of M1 (Galvão et al., 2014; Rose et al., 2014; Rocha et al., 2015; Du et al., 2016; Hosomi et al., 2016; Tosun et al., 2017). The combination of rTMS and M1 with peripheral sensory stimulation was also a promising strategy to facilitate motor recovery after stroke, more than each technique used in isolation (Celnik et al., 2009).

Few studies have modulated the S1 excitability to enhance motor function in stroke survivors. Based on the inhibitory interhemispheric connections of S1 (Eickhoff et al., 2008; Klingner et al., 2011), Meehan et al. (2011) showed that continuous theta-burst stimulation, an inhibitory form of patterned rTMS, over the contralesional S1 paired with motor training, resulted in substantial UL motor improvements. To date, no clinical trial has combined rTMS over S1 with SS. However, our preliminary findings suggest that this strategy may be promising for treating both sensory and motor disorders after stroke.

Interventions to up- or downregulate cortical excitability after stroke have targeted many clinical studies (Nowak et al., 2009; Borich et al., 2015). Approaches to reduce motor impairments by increasing S1 excitability rely on targeting sensory afferents or cortical somatosensory representations (Borich et al., 2015). In our study, combining these two approaches presumably increased the efficacy of each technique, resulting in increasing their individual effects on S1 excitability and concurrently improving somatosensory processing and M1 excitability. This could explain, at least in part, the larger improvements observed in the rTMS/SS group. Advances in neurophysiological research are necessary to determine the mechanisms underlying the benefits of combining central and peripheral sensory stimulation in neurorehabilitation. Multimodal therapies could be a more effective strategy for promoting sensory and motor recovery after stroke, allowing simultaneous access to the injured cortical network at the central and peripheral levels.

Combining rTMS with behavioral therapies, such as SS, can be useful for extending the therapeutic window, thus offering a greater opportunity for physical and occupational therapies to promote functional recovery (Bolognini et al., 2009).

It is not yet clear which neural mechanisms may be responsible for the trend observed in this study with respect to the superiority of our combined (rTMS + SS) approach. We can speculate that the same mechanism responsible for the larger effect induced by the dual use of M1 rTMS and motor therapy may likely account for the present findings. rTMS can modulate cortical excitability and induce long-term after-effects (Pascual-Leone et al., 1994). Depending on the rTMS parameters, long-term suppression or facilitation of cortical excitability can be induced; low-frequency rTMS (≤1 Hz) usually results in decreased cortical excitability (Chen et al., 1997), whereas at higher frequencies (>1 Hz), cortical excitability is generally increased. The mechanisms underlying the after-effects of rTMS are not completely understood, but they seem to involve synaptic plasticity, such as long-term potentiation or depression, as seen in the hippocampus after repeated synaptic activation (Wang et al., 1999; Hoffman and Cavus, 2002; Huang et al., 2005).

In addition, the modulation of neurotransmitter levels appears to be a contributing factor. The neurotransmitter systems involved include the GABAergic system (Donoghue et al., 1996), as well as the excitatory glutamatergic system with NMDA receptor activation (Hess and Donoghue, 1996). TMS can result in changes in endogenous neurotransmitters (GABA and glutamate) and neuromodulators (DA, NE, 5-HT, and ACh), which play key roles in regulating neuronal activity in the cerebral cortex [for review, see Hasselmo (1995)].

Similarly, learning processes promoted by behavioral training can lead to the strengthening of existing neural pathways and to new changes or adaptations, and thus, the expression of neuroplasticity (Pascual-Leone et al., 2005). Since non-invasive brain stimulation and behavioral therapies share similar mechanisms of action to induce neuroplastic changes in the human cortex, a possible conjecture is that their combined use can maximize their individual effects since the learning processes are accompanied by changes in cortical excitability and changes in synaptic efficacy.

Considering that the latter effect of NIBS is dependent on the NMDA receptor, changes in cortical excitability induced by S1 rTMS may interact with those activated by our SS protocols, reinforcing their individual effects on sensorimotor functions (Bolognini et al., 2009). This, in turn, has allowed an increase in the clinical gains in motor function, with improvements that cannot be achieved by administering rTMS or SS alone (Bolognini et al., 2009). We discuss the results of a possible increase in S1 cortical excitability with an indirect effect on motor improvement.

The participants in the control group did not show significant improvement either in the raw scores of the evaluations or in the minimal clinically important difference. However, we believe that even in the optimal phase of neuroplasticity, 2 weeks (the duration of the protocol) is a short period to observe significant improvements. It is possible that the condition of these participants may have improved over time. However, the period in which we applied the interventions and evaluated them was not sufficient for the participants in the fictitious group to show significant improvements. Thus, we believe that rTMS may favor the early opening of a neuroplastic window.

### Limitations

A major limitation of our study was the differences among the groups at baseline for most of the ICF activity/participation measures. The severity of sensory and motor impairments at baseline has certainly impacted the treatment efficacy; further investigation in more clinically homogeneous samples is required to confirm and extend the present findings. Some participants could not perform the JJT or lacked baseline data for various reasons, so they could not be included in the analyses. A small sample size is a matter that needs to be considered. Another limitation of our study was with the sham SS group. In fact, active sensory training performed with the non-paretic upper limb is not completely ineffective since it can modulate contralesional S1 excitability, thereby affecting the results. Another limitation is that our study mainly used subjective methods (i.e., clinical tests and scores) to measure upper limb function. We suggest that future studies use more objective methods to measure the functions. We also considered the lack of physiological indicators to assess cortical excitability at M1 and S1 as a limitation of this study. We suggest that future studies include assessments of motor and somatosensory-evoked potentials.

We could not control some aspects of the participants’ daily lives; for instance, some were working concurrently with therapy, while others were retired or at home. This could be a factor that interferes with the results. We suggest that future studies should better control this variable. From a theoretical point of view, the combination of various types of SS, without assessing their individual effects and how each of them interacts with S1-rTMS, renders it difficult to delineate the potential neuro-functional basis of their clinical efficacy, either when delivered as monotherapy or in conjunction with somatosensory cortical stimulation.

## Conclusion

Considering the positive trend of the clinical effects of the combined use of S1-rTMS and SS compared to each therapy alone, we conclude that combined use of SS with rTMS over S1 represents a more effective therapy for increasing sensory and motor recovery, as well as functional independence, in participants with subacute stroke. Central and peripheral sensory stimulation may represent a potential new strategy for post-stroke sensorimotor rehabilitation. Future studies are required to confirm the present preliminary evidence and determine the underlying neurofunctional basis.

## Data Availability Statement

The raw data supporting the conclusions of this article will be made available by the authors, without undue reservation.

## Ethics Statement

The studies involving human participants were reviewed and approved by local Human Research Ethics Committee Federal University of Pernambuco. The patients/participants provided their written informed consent to participate in this study.

## Author Contributions

AFZ: text design, elaboration, writing, data collection, analysis, and revision. ACRS and ABS: data collection and revision. ABRM and LSGN: data collection, analysis, and revision. NB: writing and revision. KM-S: text design, writing, and revision. All authors contributed to the article and approved the submitted version.

## Conflict of Interest

The authors declare that the research was conducted in the absence of any commercial or financial relationships that could be construed as a potential conflict of interest.

## Publisher’s Note

All claims expressed in this article are solely those of the authors and do not necessarily represent those of their affiliated organizations, or those of the publisher, the editors and the reviewers. Any product that may be evaluated in this article, or claim that may be made by its manufacturer, is not guaranteed or endorsed by the publisher.

## Footnotes

1
https://clinicaltrials.gov

2
www.randomization.com
